# Quality of Life of Donors After Living Kidney Transplantation: A Cross-Sectional Study

**DOI:** 10.3390/life15020325

**Published:** 2025-02-19

**Authors:** Chrysanthi Skalioti, Maria Smyrli, Chrysoula Beletsioti, Stathis Tsiakas, Kalliopi Vallianou, John N. Boletis, Smaragdi Marinaki

**Affiliations:** 1 Department of Nephrology and Renal Transplantation, Medical School, National and Kapodistrian University of Athens, 11527 Athens, Greece; mariasmirli3002@hotmail.com (M.S.); stathis.tsiakas@gmail.com (S.T.); kallia_harry@hotmail.com (K.V.); inboletis@gmail.com (J.N.B.); smaragdimarinaki@yahoo.com (S.M.); 2 Department of Quality Control, Research and Continuing Education, General Hospital of Nikaia “Agios Panteleimon”, 18454 Athens, Greece; chrysa510@gmail.com

**Keywords:** kidney donation, kidney donors, quality of life, questionnaire SF-36

## Abstract

Living kidney transplantation yields better outcomes for recipients than deceased donation, though it may present long-term challenges for donors. This study assessed the quality of life (QoL) of living kidney donors in Greece using the SF-36 questionnaire and examined associated sociodemographic and clinical factors. This cross-sectional study included 242 clinically stable kidney donors from Laiko General Hospital’s Kidney Donor Outpatient Clinic in Athens. Data on demographics, comorbidities, laboratory parameters, and QoL were collected. Spearman’s correlation coefficients were used to identify associations between QoL scores and influencing factors. The majority of donors were female (73.55%). Donors reported high QoL, with median PCS and MCS scores of 55.27 (49.08–57.73) and 54.17 (46.64–59.93), respectively. Male donors had higher PCS and MCS scores than females (*p* = 0.028 and *p* = 0.004). Laparoscopic nephrectomy was associated with better mental health and physical outcomes compared to open surgery (*p* < 0.001). Higher education and eGFR correlated with better PCS scores, while older age negatively affected QoL (*p* < 0.05). Living kidney donors reported a satisfactory level of overall QoL post-donation. Lower QoL scores were correlated with the female gender, older age, and open nephrectomy. These findings may further reinforce support for our current organ donation policy. Non-modifiable factors appear to influence donor quality of life. These findings reinforce the current organ donation policy while emphasizing the need for careful pre-transplant counseling and ongoing monitoring post-donation.

## 1. Introduction

Living kidney transplantation offers superior graft and patient survival outcomes for recipients compared to deceased kidney transplantation [1]. In response to the rising demand for kidney allografts, strategies have been introduced to expand the pool of living donors [2]. According to the Global Observatory on Donation and Transplantation, 39% of the 102,090 kidney transplants performed in 2022 were sourced from living donors, while in 2018, living kidney donations accounted for 36.2% of total kidney transplant activity [3]. In 2023, 10,404 individuals were active on the Euro-transplant waiting list, while 3161 kidney transplantations were performed [4]. In response to the increasing demand for kidney allografts, strategies have emerged aimed at expanding the pool of living donors. Furthermore, perioperative morbidity and mortality have decreased with the advent of minimally invasive surgical techniques [5,6,7].

Candidacy for kidney donation can have various implications for donors, including changes in family dynamics, relationships, and lifestyle. Additionally, skepticism and doubt often arise in the majority of donors regarding the pre-transplantation process, the short- and long-term risks of donation, the information provided from healthcare professionals, and renal function following nephrectomy [8].

Perioperative complications have been reported in 13–16.8% of donors in multicenter studies [9,10]. Moreover, the most common long-term complication after donation is hypertension, albeit recent data show a comparable incidence of hypertension among donors and non-donors [11,12]. The decline in kidney function and albuminuria have been observed post-donation [12,13].

Wirken and colleagues have reported that concerns about the health of recipients have been linked to the well-being of donors in the period shortly after nephrectomy [14]. Additionally, existing research suggests that the outcomes of recipients have a corresponding impact on the donor’s long-term quality of life and mental well-being [15,16]. Several studies have also examined the incidence of anxiety and depression among kidney donors, reporting varying incidence rates [17,18,19,20,21]. This risk has been observed in women, especially in cases where symptoms of anxiety and depression are present before donation [22]. Furthermore, impaired physical health has been associated with a decreased quality of life (QoL) following kidney donation [23].

On the other hand, previous studies suggest that donors do not experience a decline in QoL, with levels remaining comparable to those prior to donation [24,25,26]. Additionally, the mental and physical well-being of kidney donors appears to be similar to that of the general population [16,27]. Some authors suggest that kidney donors may experience improved QoL due to the altruistic nature of their decision to donate and the support they receive from their families [28]. Additionally, existing research indicates that positive outcomes for recipients have a beneficial effect on the donor’s quality of life and mental well-being in the long term.

However, laboratory parameters and the comorbidities of donors, as well as their impact on QoL are rarely explored in the available literature.

Given that living kidney donors account for more than 50% of the total donor population in Greece [29], examining the quality of life (QoL) status of our donors is of paramount importance.

The purpose of our study is to assess the quality of life of kidney donors at a large transplant center in Greece using the SF-36 questionnaire, and to explore its association with potential influencing factors.

## 2. Materials and Methods

### 2.1. Aim

This study aimed to explore the following: 1. evaluate the quality of life of living kidney donors (QoL); 2. investigate the factors associated with QoL status.

### 2.2. Study Design

This was a cross-sectional correlational study in living kidney donors under regular follow-up conditions at the Kidney Donor Outpatient Clinic of Laiko General Hospital in Athens, Greece.

### 2.3. Participants

Eligible participants were adult living kidney donors who visited the Kidney Donor Outpatient Clinic of Laiko General Hospital in Athens, Greece, for routine follow-up between 1 January 2023 and 1 January 2024. We used the method of convenience sampling [30]. All subjects signed informed consent after receiving detailed information. Participants were assured of the confidentiality of their responses before providing written consent, and their legal right to withhold participation was fully respected. Clinically stable kidney donors with at least 12 months of follow-up after nephrectomy and complete information on the main variables of interest were enrolled in the study. Exclusion criteria included the following: 1. an inability to understand and respond to the questionnaire; 2. clinically unstable individuals with acute disease.

The Renal Transplant Unit of Laiko General Hospital represents the largest transplant unit in Greece, performing the vast majority of living kidney transplants in the country annually. According to our practice, living kidney donors visit the outpatient clinic regularly at intervals ranging from 3 months up to 1 year, with the more frequent visits scheduled during the first year after donation. At each visit, a complete hematological and biochemical routine laboratory workup is performed. Participants’ clinical history, medications, body weight (BW), and blood pressure (BP) are also monitored and recorded at every visit.

For the purposes of this study, health-related quality of life (HRQoL) was assessed during the scheduled appointment using the Short Form (36) (SF-36) Health Survey, which, through 36 questions, examines eight different health-related parameters, namely physical functioning (PF), physical role (RP), bodily pain (BP), general health (GH), vitality (VT), social functioning (SF), emotional role (RE), and mental health (MH). These scales are summarized into two general categories: a physical component summary (PCS) and a mental component summary (MCS) [31]. The validated and normalized Greek version of SF-36 was utilized [31]. The questionnaire was completed on the day of each donor’s scheduled outpatient visit.

The study protocol was reviewed and approved by the Institutional Review Board of «Laiko» General Hospital (378/9 January 2025).

### 2.4. Data Collection

All study data for each donor were recorded during their scheduled visit in designated medical charts. We collected routine data on the sociodemographic parameters (gender, age at the time of donation, age at the time of examination, body mass index, BMI), and marital status. The level of education was categorized into 9-year compulsory, 12-year, and tertiary education. The tertiary level refers to the stage of education that follows secondary school, typically provided at public and private universities, and includes undergraduate and/or postgraduate studies. Donors were categorized as biologically related and unrelated. The first group included parents, grandparents, siblings, cousins, and uncles/aunts, namely first- and second-degree relatives. Biologically unrelated donors were spouses and individuals with a long-term relationship with the recipient, mainly friends.

Comorbidities (arterial hypertension, diabetes mellitus, DM, cardiovascular disease, CVD), smoking, and routine hematological and biochemical parameters were recorded for each subject. The Chronic Kidney Disease Epidemiology Collaboration (CKD-EPI) equation was used to calculate the estimated glomerular filtration rate (eGFR) based on serum creatinine [32]. Information on current disease history and blood pressure readings were obtained from donors.

### 2.5. Statistical Analysis

Descriptive statistics were calculated for the demographic and clinical characteristics, the SF-36 eight subscale, and two summary component scores. The normality of the distribution of continuous variables was tested with the significance of the Kolmogorov–Smirnov test, and non-normally distributed data were described using the median and interquartile range, while normally distributed data were described using the mean and standard deviation. Categorical variables were presented as frequencies and percentages.

The SF-36 subscales were calculated using a scoring algorithm, recommended by the developers, in order to code, sum, and transform the responses per dimension into a scale from 0 to 100, with higher scores indicating better health status [33]. The summary components were computed through a standardized three-step process that added together the scores from all domains with differing weightings, according to a principal component analysis procedure [33].

Mann–Whitney U tests were used to evaluate differences in the distribution of health-related quality of life scores regarding sociodemographic and clinical characteristics. Additionally, Spearman’s correlation coefficients were used to analyze the relationships between the SF-36 subscale and summary scores and demographic and clinical variables due to the non-normal distribution of SF-36 scores. The minimum required sample size for the Mann–Whitney test was calculated using G-power (with an effect size of 0.5, α = 0.05, 1 − β = 0.8, and the allocation ratio of participants in each group was 0.2) for 240 participants. Statistical analyses were performed using IBM SPSS Statistics for Windows, Version 28. Significance was set at α = 0.05, and all tests proceeded as two-tailed.

## 3. Results

### 3.1. Sociodemographic and Clinical Characteristics

**Figure 1:**
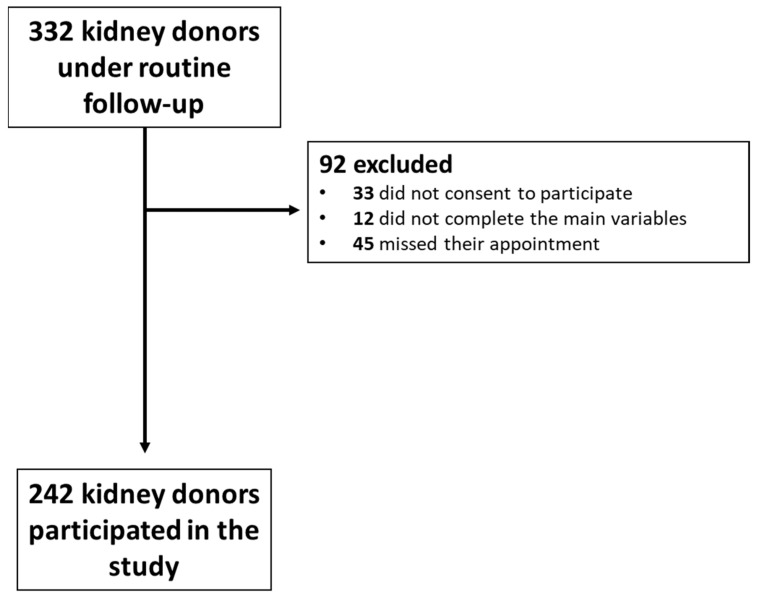
Flow chart of patient selection.

Out of a total of 332 kidney donors who had a scheduled appointment during the aforementioned period, 33 did not consent to take part in the survey, 12 were excluded as they did not complete the main variables of interest, and 45 missed the appointment (Figure 1). Thus, the final population consisted of 242 donors. Sociodemographic characteristics of the population and laboratory parameters are presented in Table 1 and Table 2, respectively. From the total cohort, 178 donors were female (73.55%), with a median age of 63.10 (55.88, 70.58) years. After a median time of 1.8 (1.07–4.1) years post-donation, the mean eGFR was 60.8 ± 13.93 mL/min/1.73 m^2^. In terms of education level, 22.31% of the participants had completed tertiary education. A donor–recipient biological relationship was reported in 75.21% of the transplants. Laparoscopic nephrectomy was recorded in 68.2% of the donors.

### 3.2. QOL Questionnaire SF-36

The QOL of our donors appears to be satisfactory, as indicated by median scores in the eight health-related subscales ranging from 84 to 100 (on a scale of 1–100). The median overall PCS and MCS scores for our donor population were 55.27 (49.08, 57.73) and 54.17 (46.64, 59.93), respectively. The highest score of 100 was observed in four categories, RP, BP, SF, and RE, followed closely by physical functioning PF at 95.00 (80.00, 100.00) (Table 3). The lowest score was observed in MH and VT.

Our analysis revealed that men had significantly higher overall median PCS and MCS scores (56.28 (52.12, 57.87) and 58.01 (51.94, 61.15, respectively)) compared to women (55.03 (48.09, 57.42) and 52.93 (45.43, 58.89), *p* = 0.028 and *p* = 0.004, respectively) (Table 4). Notably, male donors outperformed female donors in five out of the eight health-related categories, with the exception of bodily pain (BP), general health (GH), and SF, where the scores were similar between genders.

Older age at both the time of donation and examination was a negative prognostic factor of lower PF and PCS (correlation coefficient (Rs = −0.157, *p* = 0.015 and Rs = −0.196, *p* = 0.002, respectively) (Table 5).

No significant associations were found between smoking, DM, CVD, hypertension, obesity (BMI > 30), and biologically related donation with the components of the questionnaire (Table 4).

Laparoscopic nephrectomy showed a strong association with enhanced mental health (MH) (median score 88 (72, 98)) and MCS (median score 56.76 (47.40, 61.15)) compared to open surgery (median score 76 (62, 86) and 51.87 (44.85, 56.41), respectively, with a *p*-value < 0.001)). Similarly, laparoscopic surgery was correlated with better scores in the PF, GH, and VT domains (95 (80, 100), 87 (77, 97) and 80 (60, 90), respectively) compared to the open technique (90 (75, 100), 82 (71, 92) and 70 (57.50, 85), respectively), (*p* = 0.049, *p* = 0.014 and *p* = 0.029, respectively). In contrast, the laterality of nephrectomy (left or right) did not show any significant correlation with the SF-36 parameters.

Biologically related donations showed similar scores to those from unrelated donors. Furthermore, marital status was not correlated with the donor’s mental or physical well-being post-nephrectomy across all components of the SF-36 survey (Table 4).

Donors with a tertiary level of education had higher PCS median scores 56.90 (52.62, 57.88), particularly in PF (100 (90, 100)), GH (90 (79.25, 100)), and SF (100 (87.50, 100)), compared to those without tertiary education (54.89 (48.32, 57.35), 90 (75, 100), 85 (72, 96.50) and 100 (75, 100), respectively) with *p* = 0.02, *p* = 0.004, *p* = 0.027, and *p* = 0.032, respectively)) (Table 4). Furthermore, the analysis showed that a higher eGFR positively influenced donors in terms of PF and PCS (Rs = 0.134, *p* = 0.038 and Rs = 0.148, *p* = 0.022, respectively) (Table 5). Conclusions can be drawn from this.

## 4. Discussion

The aim of our study was to evaluate the quality of life of living kidney donors and to examine the effect of sociodemographic, clinical, and laboratory parameters on the level of QoL.

Kidney donors in the present study reported a satisfactory level of QoL. Physical and mental health status were numerically better than the general population of Greek adults [31].

Prior single-center and international investigations demonstrate similar results, emphasizing that the status of QoL after donation does not differ from that of the corresponding general population [17,19,21,30,31,32,33,34,35,36,37]. Mjoen and colleagues conducted a survey in Norway with the participation of 76% of kidney donors registered in the Norwegian database, who had, on average, donated 12.6 years earlier [28]. The QoL of kidney donors was comparable to that of the general population. In a large cross-sectional multicenter study with a mean follow-up of 17 years, Gross and colleagues found that over 80% of the participants reported their health status to be average or better compared to the national norm [27]. The reported results applied to both White and African American donors.

In a recent meta-analysis of nine studies, Liu and colleagues [10] examined the impact of donation on the QoL of kidney donors in China. The authors concluded that, although living kidney donors experienced more bodily pain than the general population, they demonstrated superior scores in the GH dimension. No significant difference was observed between donors and the general population in terms of PF and RP. QoL in living kidney donors was higher than that of the general population in the MCS category as well.

Although no direct comparison has been made between our cohort and the Greek population, the scores in our cohort surpass those of the general population in all SF-36 domains. The study by Pappa and colleagues that validated the SF-36 questionnaire in a population-based sample was published about two decades ago [31]. Thus, it could be argued that a comparison with the Greek norm may not be relevant after so many years. However, it is important to remember that over the past 13 years, Greece has not only faced the SARS-CoV-2 pandemic but also a severe economic crisis, which, according to a recent study by Yfantopoulos and colleagues, has had a significant negative impact on the quality of life [32].

According to our results, the MH and VT components had the lowest scores. However, these scores were still higher than those previously reported for the Greek norm [31].

Psychological factors such as anxiety and depression negatively affect mental health. Additionally, fatigue and loss of energy have been reported after kidney donation [15].

In the present study, male gender and laparoscopic nephrectomy were identified as factors positively associated with the quality of life of donors.

The male gender was associated with greater PCS and MCS scores post-donation compared to females, which is a finding also observed in previous studies [15,21,26]. It is important to note that Pappa E and colleagues reported lower values across all SF-36 parameters in women compared to men when validating the Greek SF-36 Health Survey [31]. On the other hand, the investigation of fatigue in different populations has yielded conflicting results on gender discrepancies [38,39]. Therefore, we believe that this observation should not influence our center’s donation policy practices.

Laparoscopic living donor nephrectomy is the standard of care, offering reduced hospitalization time, less pain, and an earlier return to daily activities compared with the open technique [40,41]. In our center, laparoscopic surgery supplanted open donor nephrectomy in October 2018. We have previously demonstrated the favorable effect of laparoscopic compared with open nephrectomy on the QoL of our kidney donors, which is a finding that has been confirmed in the current study [42].

Old age at the time of donation and at the time of examination was a negative predictor of lower physical functioning, which resulted in a reduced PCS level compared to younger individuals. Previous studies have underscored that older individuals require longer time to recover physical functioning. Nonetheless, in the long term, this difference seems to be mitigated [27,28,43]. Consistent with previous research, the components of mental health were not correlated with age, possibly related to the altruistic nature of donation that exerts a positive effect on emotional well-being [26,27]. Hence, older subjects should not be precluded from donation based solely on age.

The level of educational attainment was found to be significantly correlated with the domains of physical function, as well as social functioning. Given that the socioeconomic status appears to be closely associated with self-rated health [32], donors with higher educational levels were expected to estimate their health more favorably than those with lower education [27,28,43]. Interestingly, the RELIVE study revealed no effect of educational achievements on the QoL of African American donors post-donation [27].

In this study, we recorded routine hematological and biochemical parameters. Our evaluation showed that overall QoL was not significantly affected by these factors, with the exception of the association of better eGFR with the PF domain. This is in keeping with a recent study from Korea, which revealed that objective data were not associated with QoL status [44]. On the other hand, a retrospective study of 508 kidney donors, predominantly female (79.45%), revealed that good renal function post-donation was associated with higher quality of life, especially when donors were also informed about their recipient’s favorable kidney outcome [15]. This highlights the importance of satisfactory graft function for the well-being of living donors.

The impact of comorbidities and smoking on physical and mental health was examined. We observed no relation between chronic diseases or smoking and QoL. Several explanations may account for these findings. First, this could be considered the result of a proper donor evaluation, which is a complex process that aims to select those individuals with no or minor comorbidities in order to ensure optimal donor and recipient outcomes. Second, comorbidities like DM and CVD were uncommon among participants, which made it challenging to detect an effect on QoL. Hypertension was the most frequent disorder reported in 24.38% of participants. However, this rate is lower than previously reported among similar cohorts [11,21], as well as in the Greek population [45]. Third, one could argue that the time since the donation was relatively short, and the incidence of comorbidities may increase in the long term, potentially impacting QoL. Notwithstanding this, Alayoobi and colleagues did not find an association between comorbidities and QoL in a small group of individuals with a long follow-up of 111.5 months after donation [28].

Donor obesity prior to transplantation has received attention in recent years [46,47,48,49]. According to the RELIVE study, obesity at the time of donation is an independent risk factor for physical impairment among 2455 subjects between 5 and 48 years after the nephrectomy [27]. On the other hand, excess body weight has not been associated with the MCS scores [27,28]. Nonetheless, no data were available on the impact of post-donation BMI on QoL. Although we do not report data on pre-transplant obesity, we recorded BMI after nephrectomy, which did not appear to influence QoL.

In line with the existing literature, we found that the relationship of the donor to the recipient did not influence the donor’s QoL after the nephrectomy [35,44]. Our cohort did not include altruistic, non-directed donors. In Greece, non-directed donation has only been recently implemented for cases involving recipients with high immunologic risk who cannot receive a transplant from their directed living donor. It is noteworthy that non-directed donors exhibit a high level of mental and physical well-being comparable to [25,50] or superior [51] to directed donors. Despite the fact that 75.21% of our donors were biologically related to their recipient, we believe that this finding underscores the consciousness of the decision to donate in this cohort.

Notably, we observed high scores in several domains of the questionnaire shortly after donation. We consider this an important finding, suggesting that donation did not negatively impact the donors’ well-being.

Potential donors are characterized by their willingness to donate and display excellent general health and vitality superior to the general population [52]. When assessing the well-being of living kidney donors, it would be interesting to evaluate data from actual donors compared to candidates who do not proceed with the donation process, which is a topic that has yet to be explored.

This study has limitations and strengths. Firstly, the cross-sectional design of the study did not allow for the detection of alterations in the QoL of kidney donors over time. Moreover, comparing the differences between baseline and follow-up measurements offers more meaningful insights than relying solely on absolute numbers at follow-up. However, baseline data for QoL were lacking in our population. We did not collect data, retrospectively, since this practice may be influenced by recall bias. Secondly, the study is prone to sampling bias, as participant recruitment took place over a specific time period. Convenience sampling was followed. Subjects with appointments within a specific time frame were included, provided that they were willing to participate, thus excluding the rest of the donor cohort who were not scheduled during that period [30]. Thirdly, psychiatric or psychological factors that could impact QoL and mental health were not recorded in the current study. Fourthly, the recipient outcomes that appeared to affect donors’ QoL were not documented in the current study. Fifthly, the results should be interpreted with caution in terms of generalizability because the participants were kidney donors followed at a single center. Our sample is adequate to detect statistically significant differences among all groups apart from DM-, CVD-, educational level- and marital status- related groups. Additionally, the non-normal distribution of HRQL scores could mean that our sample is not representative and that the applicability of our results to the whole population could be questioned. However, an important strength of this study is that all individuals were monitored by the same surgical and nephrology teams at the largest transplant center in the country. Thus, the study cohort can be regarded as a representative national sample.

To the best of the authors’ knowledge, this is the first study to analyze real-world data regarding kidney donors in our country. With a significant number of living donor transplants performed each year, our objective was to contribute to ongoing research and help shape a safe donation policy.

Kidney donation candidates are always evaluated by a psychiatrist prior to donation. Nonetheless, we believe that regular psychological counseling during the pre-transplant period may prepare individuals for any emotional challenges before and after nephrectomy. Counseling services should be tailored to the individual health status of the donor, taking into account factors such as age, gender, and comorbidities. Furthermore, providing access to counseling services can address any anxiety, depression, or post-surgical stress that may arise. Psychological well-being after donation may be improved through participation in donor support groups and regular medical monitoring for the prompt identification of health issues.

In conclusion, living kidney donors in Greece demonstrate a satisfactory level of quality of life. Non-modifiable factors, like gender and age, influence the status of donor well-being, indicating that kidney donation is generally a safe procedure in terms of physical and mental functioning after the surgery.

## Figures and Tables

**Table 1:** Sociodemographic and clinical characteristics of the total study cohort.

All Participants N: 242	
Male, N (%)	64 (26.45)
Female, N (%)	178 (73.55)
BMI ≥ 30, N (%)	52 (21.49)
Age at donation, median [IQR]	60.70 [52.55, 67.20]
Age at examination, median [IQR]	63.10 [55.88, 70.58]
Time between donation and examination	1.8 [1.07–4.1]
Comorbidities	
HΤΝ, N (%)	59 (24.38)
DM, N (%)	9 (3.72)
CVD, N (%)	4 (1.65)
Smoking, N (%)	9(3.72)
Lap-Nephrectomy, N (%)	165 (68.18)
Left kidney removed, N (%)	214 (88.43)
Biologically related, N (%)	182 (75.21)
Education level	
Tertiary, N (%)	54 (22.31)
12-year (Lyceum), N (%)	76 (31.4)
9-year (Compulsory), N (%)	112 (46.28)
Marital status	
Married, N (%)	224 (92.56)
Single, N (%)	3 (1.24)
Divorced or widowed, N (%)	15 (6.19)
SBP, median [IQR]	120.00 (116.50, 130.00)
DBP, median [IQR]	75.00 (70.00, 80.00)

Normally distributed data were described by their mean and standard deviation; otherwise, the median and interquartile range were reported. IQR: interquartile range.

**Table 2:** Biochemical characteristics of the total study cohort.

Parameter	Median [IQR]/Mean (SD)	Normal Range
Hemoglobulin (g/dL)	13.60 (1.17)	13.5–18.0
Hematocrit (%)	41.02 (3.19)	40–54%
White Blood Cells (Κ/μL)	6455.00 [5695.00, 7536.50]	4.50–11.00
Platelets (Κ/μL)	249,000.00 [216, 500.00, 294, 250.00]	140–440
Urea (mg/dL)	39.50 [33.00, 45.25]	16.6–48.5
Creatinine (mg/dL)	1.07 [0.95, 1.22]	0.67–1.17
eGFR (CKD-EPI)	60.80 (13.93)	
Glucose (mg/dL)	95.00 [88.00, 101.00]	70–100
Potassium (mmol/L)	4.70 [4.40, 4.90]	3.5–5
Sodium (mmol/L)	140.00 [139.00, 142.00]	135–145
UricAcid (mg/dL)	5.64 (1.39)	3.4–7.0
Vitamin D (ng/mL)	28.46 (10.58)	>30
Urine Protein (mg/24 h)	93.00 [69.00, 133.00]	<150

**Table 3:** SF-36 quality of life scores in kidney donors. PCS = physical component summary score; MCS = mental component summary score; PF = physical functioning; RP = role physical; BP = bodily pain; GH = general health; VT = vitality; SF = social functioning; RE = role emotional; MH = mental health.

Parameter	Median (Interquartile Range)
PCS	55.27 (49.08, 57.73)
MCS	54.17 (46.64, 59.93)
PF	95.00 (80.00, 100.00)
RP	100.00 (75.00, 100.00)
BP	100.00 (68.50, 100.00)
GH	87.00 (75.00, 97.00)
VT	80.00 (60.00, 90.00)
SF	100.00 (75.00, 100.00)
RE	100.00 (66.67, 100.00)
MH	84.00 (68.00, 96.00)

**Table 4:** Results of Mann–Whitney U-tests for SF-36 subscale and component scores by sociodemographic or clinical-related group. Probability values of *p* < 0.05 were considered statistically significant in all comparisons and are in bold. PCS = physical component summary score; MCS = mental component summary score; PF = physical functioning; RP = role physical; BP = bodily pain; GH = general health; VT = vitality; SF = social functioning; RE = role emotional; MH = mental health; DM = diabetes mellitus. CVD = cardiovascular disease.

	PF	RP	BP	GH	VT	SF	RE	MH	PCS	MCS
	Median (IQR)	Median (IQR)	Median (IQR)	Median (IQR)	Median (IQR)	Median (IQR)	Median (IQR)	Median (IQR)	Median (IQR)	Median (IQR)
Male	97.50 (85, 100)	100 (100, 100)	100 (80, 100)	87 (77, 97)	80 (70, 90)	100 (75, 100)	100 (100, 100)	88 (77, 96)	56.28 (52.12, 57.87)	58.01 (51.94, 61.15)
Female	90 (75, 100)	100 (50, 100)	100 (62, 100)	87 (72, 97)	75 (60, 90)	100 (75, 100)	100 (66.67, 100)	80 (64, 92)	55.03 (48.09, 57.42)	52.93 (45.43, 58.89
*p*-value	**0.027**	**0.005**	0.072	0.216	**0.020**	0.055	**0.002**	**0.014**	**0.028**	**0.004**
BMI ≤ 29.9	95 (80, 100)	100 (75, 100)	100 (72, 100)	87 (75, 97)	80 (60, 90)	100 (75, 100)	100 (66.67, 100)	84 (68, 96)	55.49 (49.76, 57.87)	54.60 (46.86, 60.15)
BMI > 30	90 (71.25, 100)	100 (50, 100)	100 (62, 100)	82 (75, 92)	72.5 (56.25, 88.75)	100 (62.5, 100)	100 (66.67, 100)	78 (64, 92)	55.02 (46.58, 57.21)	52.82 (45.16, 58.86)
*p*-value	0.106	0.523	0.463	0.374	0.138	0.603	0.189	0.180	0.165	0.301
DM yes	85 (67.50, 100)	100 (37.50, 100)	74 (51.50, 100)	87 (68.50, 98.50)	75 (52.50, 92.50)	87.5 (62.50, 100)	100 (16.67, 100)	76 (56, 82)	55.28 (45.47, 56.32	52.54 (39.45, 56.99)
DM no	95 (80, 100)	100 (75, 100)	100 (70, 100)	87 (75, 97)	80 (60, 90)	100 (75, 100)	100 (66.67, 100)	84 (68, 96)	55.26 (49.20, 57.79)	54.43 (46.66, 59.93)
*p*-value	0.531	0.784	0.139	0.889	0.811	0.344	0.562	0.164	0.457	0.325
CVD yes	87.50 (81.25, 97.50)	100 (25, 100)	100 (71.50, 100)	89.50 (76.75, 97)	82.50 (65, 92.50)	100 (100, 100)	100 (50, 100)	82 (73, 94)	56.06 (47.07, 56.39)	56.99 (49.99, 59.93)
CVD no	95 (80, 100)	100 (75, 100)	100 (68.5, 100)	87 (75, 97)	80 (60, 90)	100 (75, 100)	100 (66.67, 100)	84 (68, 96)	55.25 (49.08, 57.77)	54.05 (46.10, 59.93
*p*-value	0.725	0.958	0.503	0.742	0.602	0.100	0.866	0.888	0.919	0.576
Laparoscopic surgery	95 (80, 100)	100 (75, 100)	100 (70, 100)	87 (77, 97)	80 (60, 90)	100 (75, 100)	100 (83.33, 100)	88 (72, 98)	55.40 (49.04, 57.87)	56.76 (47.40, 61.15)
Open surgery	90 (75, 100)	100 (62.50, 100)	100 (62. 100)	82 (71, 92)	70 (57.50, 85)	87.50 (75, 100)	100 (33.33, 100)	76 (62, 86)	55.24 (48.97, 57.27)	51.87 (44.85, 56.41)
*p*-value	**0.049**	0.423	0.445	**0.014**	**0.029**	0.102	0.105	**<0.001**	0.576	**<0.001**
Tertiary level of education	100 (90, 100)	100 (100, 100)	100 (74, 100)	90 (79.25, 100)	80 (60, 90)	100 (87.50, 100)	100 (100, 100)	86 (72, 96)	56.90 (52.62, 57.88)	55.29 (48.88, 59.95)
Other level	90 (75, 100)	100 (75, 100)	100 (62, 100)	85 (72, 96.50)	77.5 (60, 90)	100 (75, 100)	100 (66.67, 100)	80 (68, 96)	54.89 (48.32, 57.35)	53.46 (45.56, 59.91)
*p*-value	**0.004**	**0.033**	0.141	**0.027**	0.519	**0.032**	0.156	0.207	**0.002**	0.327
Married	95 (80, 100)	100 (75, 100)	100 (65.50, 100)	87 (75, 97)	80 (60, 90	100 (75, 100)	100 (66.67, 100)	84 (68, 96)	55.22 (49.04, 57.70)	54.17 (46.46, 59.82)
Single or Widowed	92.50 (73.75, 100)	100 (93.75, 100)	100 (68.25, 100)	87 (69.50, 100)	70 (55, 100)	100 (75, 100)	100 (0, 100)	80 (59, 100)	56.18 (49.84, 57.87)	53.92 (46.40, 62.14)
*p*-value	0.846	0.436	0.601	0.516	0.978	0.333	0.673	0.919	0.366	0.947
Biologically related yes	95 (80, 100)	100 (75, 100)	100 (68, 100)	87 (75, 97)	80 (60, 90)	100 (75, 100)	100 (66.67, 100)	84 (68, 96)	55.22 (48.83, 57.87)	54.05 (46.65, 59.92)
Biologically related no	90 (80, 100)	100 (75, 100)	100 (65.50, 100)	87 (72, 97)	77.5 (60, 88.75)	100 (75, 100)	100 (66.67, 100)	84 (65, 95)	55.34 (50.08, 57.21)	55.34 (50.08, 57.21)
*p*-value	0.795	0.86	0.876	0.98	0.603	0.967	0.901	0.576	0.769	0.913

**Table 5:** Spearman correlation coefficients between SF-36 scores and several demographic and clinical parameters. Probability values of *p* < 0.05 were considered statistically significant (in bold). PCS = physical component summary score; MCS = mental component summary score; PF = physical functioning; RP = role physical; BP = bodily pain; GH = general health; VT = vitality; SF = social functioning; RE = role emotional; MH = mental health; BMI = body mass index; SBP = systolic blood pressure; DBP = diastolic blood pressure; HGB = hemoglobulin; eGFR = estimated glomerular filtration rate.

		PF	RP	BP	GH	VT	SF	RE	MH	PCS	MCS
Age at donation	Correlation Coefficient	−0.110	−0.064	−0.051	−0.078	0.043	0.009	−0.085	**0.017**	−0.157 *	**0.017**
	Sig. (2-tailed)	0.088	0.319	0.430	0.225	0.502	0.891	0.187	0.790	**0.015**	0.789
Age at examination	Correlation Coefficient	−0.160 *	−0.120	−0.072	−0.097	0.017	−0.024	−0.119	−0.023	−0.196 **	−0.010
	Sig. (2-tailed)	**0.013**	0.061	0.266	0.134	0.794	0.711	0.064	0.725	**0.002**	0.873
Time between donation and examination	Correlation Coefficient	0.023	0.026	0.089	0.075	0.049	0.037	0.034	0.009	0.045	0.019
	Sig. (2-tailed)	0.723	0.686	0.165	0.243	0.451	0.568	0.599	0.894	0.490	0.772
BMI	Correlation Coefficient	−0.103	−0.066	−0.046	−0.002	−0.036	0.028	−0.096	−0.041	−0.081	−0.021
	Sig. (2-tailed)	0.112	0.306	0.474	0.970	0.577	0.662	0.138	0.527	0.209	0.746
SBP	Correlation Coefficient	−0.029	0.064	0.072	−0.020	−0.043	0.101	0.082	−0.004	−0.009	0.058
	Sig. (2-tailed)	0.649	0.318	0.263	0.762	0.510	0.118	0.202	0.945	0.887	0.372
DBP	Correlation Coefficient	−0.082	−0.029	0.036	−0.030	−0.062	0.028	0.020	−0.056	−0.082	−0.014
	Sig. (2-tailed)	0.203	0.655	0.576	0.646	0.340	0.662	0.751	0.385	0.206	0.824
HGB	Correlation Coefficient	0.052	0.075	0.111	−0.067	0.007	−0.016	0.101	−0.027	0.096	0.005
	Sig. (2-tailed)	0.422	0.245	0.085	0.302	0.912	0.810	0.119	0.672	0.139	0.938
CREA	Correlation Coefficient	0.020	0.109	0.025	−0.010	0.092	0.061	0.080	0.099	0.010	0.101
	Sig. (2-tailed)	0.752	0.090	0.699	0.874	0.152	0.346	0.216	0.123	0.875	0.118
eGFR CKD EPI	Correlation Coefficient	0.134 *	0.045	0.064	0.082	0.002	0.033	0.077	0.004	0.148 *	0.014
	Sig. (2-tailed)	**0.038**	0.489	0.320	0.204	0.979	0.615	0.235	0.951	**0.022**	0.834
Proteinuria/24 h	Correlation Coefficient	−0.071	0.030	−0.115	0.027	−0.024	0.040	0.007	−0.063	−0.040	−0.019
	Sig. (2-tailed)	0.289	0.657	0.084	0.683	0.722	0.555	0.912	0.349	0.548	0.775

** correlation is significant at the 0.01 level (2-tailed); * correlation is significant at the 0.05 level (2-tailed).

## Data Availability

The data supporting the findings of this study can be made available upon request.

## Abbreviations

The following abbreviations are used in this manuscript:

QoL	Quality of life
BW	Body weight
BP	Blood pressure
HRQoL	Health-related quality of life
PF	Physical functioning
RP	Role physical
BP	Bodily pain
GH	General health
VT	Vitality
SF	Social functioning
ER	Emotional role
MH	Mental health
PCS	Physical component summary
MCS	Mental component summary
BMI	Body mass index
DM	Diabetes mellitus
CVD	Cardiovascular disease
CKD-EPI	Chronic Kidney Disease Epidemiology Collaboration
EGFR	Estimated glomerular filtration rate
IR	Interquartile range
SBP	Systolic blood pressure
DBP	Diastolic blood pressure
